# Experimental Analysis of Various Blockage Performance for LiDAR Sensor Cleaning Evaluation

**DOI:** 10.3390/s23052752

**Published:** 2023-03-02

**Authors:** SungHo Son, WoongSu Lee, HyunGi Jung, JungKi Lee, ChaRyung Kim, HyunWoo Lee, SeoungWoo Cho, JeongAh Jang, Michael Lee, Han-Cheol Ryu

**Affiliations:** 1 Department of Future Vehicle Research, Korea Automobile Testing and Research Institute, Hwaseong 18247, Republic of Korea; 2 Department of Convergence Science, University of Sahmyook, Seoul 01795, Republic of Korea; 3 TOD Based Transportation Research Center, University of Ajou, Suwon 16499, Republic of Korea; 4 Department of Business Intelligence and Analytics, Legacy.com, 230 W Monroe Ste 400, Chicago, IL 60606, USA

**Keywords:** blockage, LiDAR, viscosity, Arizona dust, JIS, KTD, kaolin, Portland cement, fake poop, cricket

## Abstract

Autonomous driving includes recognition, judgment, and control technologies, and is implemented using sensors such as cameras, LiDAR, and radar. However, recognition sensors are exposed to the outside environment and their performance may deteriorate because of the presence of substances that interfere with vision, such as dust, bird droppings, and insects, during operation. Research on sensor cleaning technology to solve this performance degradation has been limited. This study used various types and concentrations of blockage and dryness to demonstrate approaches to the evaluation of cleaning rates for selected conditions that afford satisfactory results. To determine the effectiveness of washing, the study used the following criteria: washer, 0.5 bar/s and air, 2 bar/s, with 3.5 g being used three times to test the LiDAR window. The study found that blockage, concentration, and dryness are the most important factors, and in that order. Additionally, the study compared new forms of blockage, such as those caused by dust, bird droppings, and insects, with standard dust that was used as a control to evaluate the performance of the new blockage types. The results of this study can be used to conduct various sensor cleaning tests and ensure their reliability and economic feasibility.

## 1. Introduction

Most of the latest automobiles are equipped with advanced driver assistance systems (ADAS), and manufacturers aim to commercialize the Society of Automotive Engineers (SAE) level four autonomous vehicles, which are fully autonomous beyond semi-autonomous driving (SAE levels two and three). Therefore, manufacturers have been conducting various studies to investigate the response to non-standard objects (e.g., traffic signals, unpaved roads) through the convergence of vehicles and infrastructure, including various new technologies, infrastructure, systems, and autonomous driving safety.

In non-autonomous vehicles, the driver is the main body responsible for collecting information, using their eyes. However, in SAE levels four and above, instead of the driver, the vehicle collects driving data using sensors such as cameras, LiDAR, and radar.

The covering material of the sensor responsible for collecting data for autonomous vehicles also influences the sensitivity of the sensor [1]. Concerns regarding the safety of autonomous driving have often been highlighted [2,3] owing to sensor performance degradation caused by materials that cover the sensor surface directly (hereafter referred to as blockages). The cognitive sensor for an autonomous vehicle is attached to the windshield wiper system of a non-autonomous vehicle [4,5,6]. Thus, it is considerably smaller than the front glass of the car. Therefore, even a minor blockage, such as a flying insect, may significantly affect sensor performance [7]. Autonomous vehicles below SAE level three include a feature that alerts drivers, such that they may fix the issue themselves or go to a safe area of the road to intervene and protect the sensor against the blockage. However, level four and above autonomous vehicles require sensor cleaning technology that can protect sensor performance from blockages on the sensor surface while driving [8]. Recently, various firms have been working on developing sensor cleaning technologies [9]; however, they follow their own dust recipes instead of an established standard. Therefore, there is a constant demand for dust recipes and standardized dust for sensor cleaning performance evaluation under laboratory conditions similar to bird droppings and insects observed on real roads.

This study does not use standard dust and concentrations but instead uses new blockage types at various concentrations bombarded onto the sensor’s surface to hinder its functions. Additionally, new experimental methods were applied by spraying the blockage to assess cleaning rates (the percentage of dust remaining after the dust is removed) under dry and non-dry conditions. 

Five different types of dust [10,11], manufactured in laboratory environments and based on various recipes, were used in conjunction with blockages that could be encountered on roads, such as bird droppings and insects, to assess whether they were similar in effect. Using the results of this study, researchers may be able to develop blockages with the desired performance by varying the dust, concentration, and drying conditions in the laboratory environment. Blockages similar to those in the actual environment that are due to agents such as insects and bird droppings, may thus be developed. The results of this study with respect to the different types of dust and their concentrations and dryness were used to realize the desired blockage effects. The experiment of the LiDAR sensor, cleaning techniques, and blockage trends are discussed below. 

### 1.1. Prior Research on LiDAR Sensor Blockage

LiDAR is a technique that uses light with a wavelength of 905 nm to detect objects. Various efforts [12,13,14,15] have been made to overcome the limitations of sensor sensitivity with technology. However, as objects are sensed by light, the blockage of the sensor surface has a significant impact on how effectively they are detected [16]. To investigate how, according to the contamination state of the sensor cover, dust impacts the degradation of LiDAR performance in an automotive environment, such as a change in the maximum detection distance of the sensor, a study was performed using dust on the surface of a LiDAR window. A study on the laser transmittance according to the level of dust buildup was conducted. Additionally, a comparison of the light transmittance according to the layer on which particular dust is deposited on the LiDAR cover and a study on the sensor sensitivity impact analysis according to haze, fog, and rainfall between the sensor and the target object [17,18,19,20,21,22,23,24,25,26,27] were also performed. To simulate the blockage that may come into contact with the sensor surface on a real road, a plastic sample used as a LiDAR cover was installed on a car grill. The impact on the transmission and reflection properties of the sensor cover was subsequently investigated [28]. Furthermore, research has been conducted on the accuracy of distance measurements, the impact on LiDAR transmission and reception rates [29], and different cleaning techniques for different sensor types [30,31].

### 1.2. Cleaning Technology

Regarding the autonomous driving sensor cleaning technology, dlhBowles [32], Ficosa [33], Valeo [34], and Continental [35] applied the cleaning system to the cameras. For LiDAR, Waymo and Valeo [34] applied the cleaning system. Sensor cleaning methods typically use a washer by default. However, typical methods of removing residue after the washer include air, wiper, and electric types.

#### 1.2.1. Air Type

**Figure 1:**
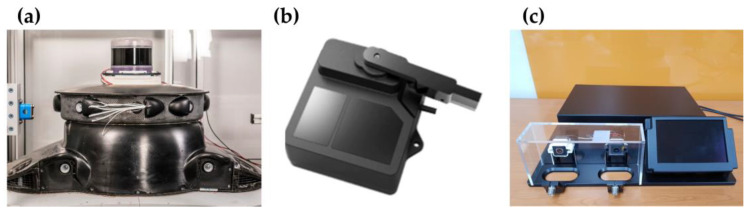
Type of sensor cleaning (**a**) Air-type cleaning system; (**b**) wiper-type cleaning system; (**c**) electric-type (piezo, electrowetting) cleaning system.

Figure 1a shows an air-type cleaning system. Air-type cleaning is the most representative method of sensor cleaning, in which a high-pressure air spray blows away the residues, removing blockages on the camera surface of the LiDAR window. The experiments in this study utilized a commonly used sensor cleaning system with a washer and air sprays.

#### 1.2.2. Wiper Type

A wiper-type cleaning system (Figure 1b) can remove the residual blockage that is not removed by the washer alone by using a wiper to make direct contact with the corresponding blockage. It is most effective in removing blockage; however, the initial installation cost, aesthetics, and durability need to be discussed.

#### 1.2.3. Electric Type

Figure 1c shows that piezo and electrowetting methods are typical examples. Piezo applies electricity to a piezoelectric element and utilizes the physical vibration method of the element to drop residual water droplets. Electrowetting is a method of removing residual water droplets by changing their shape through the application of voltage. Although it has the advantage of requiring only elements and electricity supply without the need for a complex system setup, it poses a durability problem due to damage to the exterior wire.

### 1.3. Blockage Trends

Studies on dust, bird droppings, insects, and related materials as blockages that can interfere with vision and can be experienced while driving due to direct contact with the sensor were investigated.

#### 1.3.1. Dust Class

Representative examples of dust mainly used as ISO 21103-1 test dust include ARI [36] and JIS. These dusts are classified into several categories according to particle size. ARI A2 Fine and JIS class 8, which are composed of dust of sizes 75–80 μm or less, are used in automobile window washer devices. Moreover, China test dust is also available, which is a mixture of JIS and SiO_2_ at a ratio of 1:1. The properties of the Republic of Korea test dust (KTD) and other components that are supplied in Republic of Korea are shown in Table 1.

#### 1.3.2. Bird Droppings

**Figure 2:**
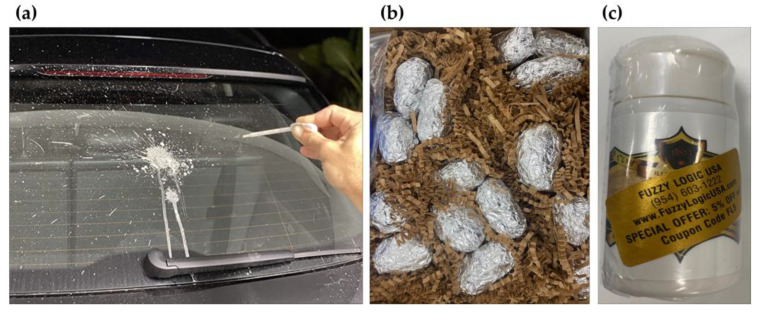
Type of bird droppings (**a**) Fake poop, (**b**) owl droppings, (**c**) UGUISU.

Figure 2 shows the representative bird droppings available for purchase, including artificial pigeon excrement [37] and owl droppings containing various components consumed by owls. Furthermore, refined whistleblower powders that are used for skin care are also available. Actual droppings were not sampled for health reasons.

#### 1.3.3. Insects

Insects are refined materials used to express contamination of the sensor surface caused by insects that rush at the light during night driving. In fact, crickets used as insect food are available for purchase according to their size and type. In addition, unspecified flying insects exist, which can be collected outdoors. In this study, crickets were purchased for use in the insect field of the tests. 

## 2. Research Equipment and Materials

### 2.1. Experimental Environment Configuration

**Figure 3:**
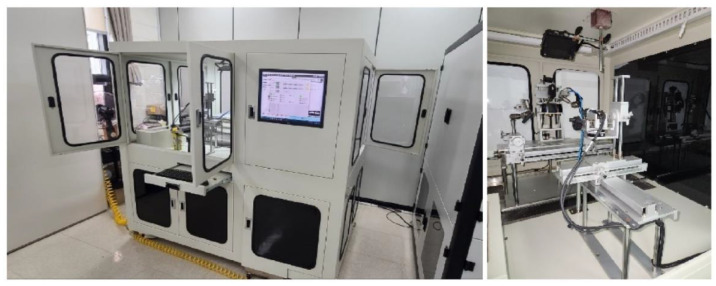
Blockage performance evaluation device.

Figure 3 shows the customized test equipment for this study. It consists of a fixed part that can fix sensors and window samples, a cleaning part that cleans with washers and air, a collector part that collects data from experiment results, and a controller part that can control experiments. In addition, a sink, an airflow pump, and a drain tank are installed. (Part name—manufacturer: nozzle—MiSUMi (511, Yeongdong-daero, Gangnam-gu, Seoul, Republic of Korea); vision camera—CREVIS (29-4, Gigok-ro, Giheung-gu, Yongin-si, Gyeonggi-do, Republic of Korea); compressor—Wabco (23, Cheongbuksandan-ro, Cheongbuk-eup, Pyeongtaek-si, Gyeonggi-do, Republic of Korea); SMPS—Mean Well; arm jig—MiSUMi; production—Soonho Technology (109, Wauan-gil, Bongdam-eup, Hwaseong-si, Gyeonggi-do, Republic of Korea)).

#### 2.1.1. Fixed Part

**Figure 4:**
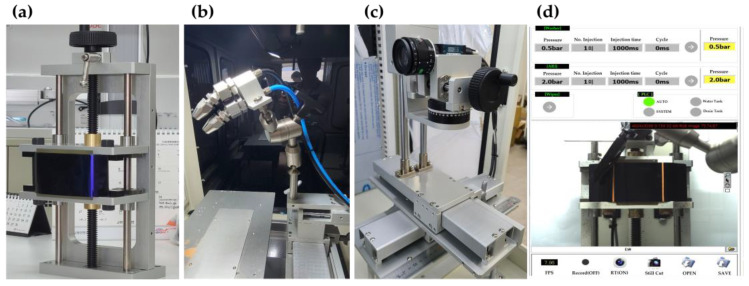
(**a**) Sample fixed part, (**b**) cleaning part, (**c**) data collector part, and (**d**) controller part.

In Figure 4a, the fixed part exhibits a shape in which the upper and lower parts are simultaneously engaged around the central point. The part in contact with the sample surface is made of urethane material to minimize damage and is designed to operate in all axial directions.

#### 2.1.2. Cleaning Part

In Figure 4b, the cleaning part comprises two sets, with a washer nozzle and an air nozzle installed in each set. Fixed installation in all directions is possible by applying the fixed part as a joint. Moreover, the position and angle of the washer and air unit can be adjusted separately.

#### 2.1.3. Collector Part

In Figure 4c, a camera capable of capturing high-definition images is installed in the collector part. Real-time videos and photographs can be acquired according to the settings. The file is automatically saved in the built-in server and is designed to operate in all axial directions.

#### 2.1.4. Controller Part

The controller part is capable of remote-controlled real-time data storage and automatic control that sets and automatically executes the experiment sequence.

### 2.2. Blockage Reproduction Device

#### 2.2.1. Dust Injector

**Figure 5:**
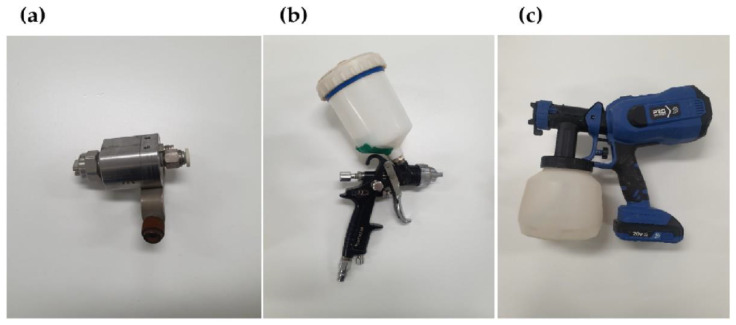
(**a**) Spray system, (**b**) gravity spray gun, and (**c**) automatic injection gun.

The spray system (manufacturer: Spraysystem(45, Hambangmoe-ro 377beon-gil, Namdong-gu, Incheon, Republic of Korea), model name: AAB10000AUH) shown in Figure 5a enables the precise spraying of fluid through precise control of the spray nozzle. Most importantly, the spray controller can control the dust injection time, duty cycle, and quantitative dust injection. However, spraying a fluid with a concentration of less than 30% is recommended because dust with relatively large particles frequently clogs the nozzle. 

The gravity spray device (manufacturer: Bluetech (100, Dalseong-ro, Jung-gu, Daegu, Republic of Korea), model name: H827) shown in Figure 5b can spray fluid using air pressure. The fluid container is located at the upper part of the spray gun to compensate for the problem of sedimentation of fluid (dust) during spraying. Therefore, it is advantageous in so far as it is feasible to evenly spray according to the ratio of the mixed fluid. Furthermore, it has the feature of evenly spraying fluid with relatively large particles. However, as it is a passive type, it cannot perform a constant quantity of spray on its own. 

The automatic injection device (manufacturer: Prostormer (Level 2, Building 2, 169 Zhen Jiang Rd., Jiangbei district, Ningbo, Zhejiang, China), model name: PTHT264), shown in Figure 4c is a rechargeable electric sprayer, which has the advantage of being able to set the nozzle type, spray pattern, and spray angle to suit the user’s needs. However, it causes the phenomenon wherein the nozzle is clogged by dust with large particles.

In this study, the three injectors were tested with various fluids. The gravity spray gun was used, which does not cause clogging when spraying up to 50% concentration. The spray used an air pressure of 6 bar with a vertical distance of approximately 30 cm to the LiDAR window, which is the dust distribution target. The test for constant quantity spray was carried out at 3.5 g by measuring the weight of the sample before and after spraying with an electronic balance.

#### 2.2.2. Insect Launcher

**Figure 6:**
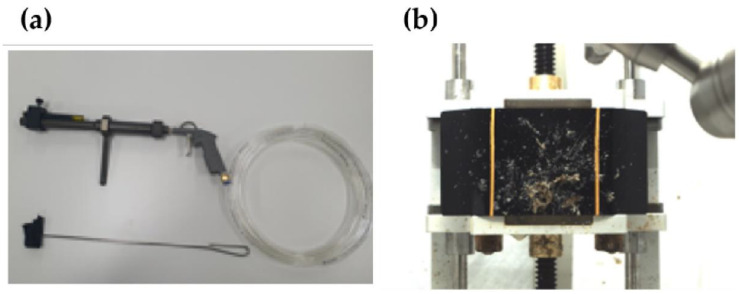
(**a**) Insect air gun; (**b**) after insect impact.

The insect gun (Figure 6a) was designed and manufactured in the form of a gun to improve mobility and accuracy (manufacturer: BMS TECH (1025-5, Gyeonggi-daero, Hwaseong-si, Gyeonggi-do, Republic of Korea)). The rate of firing can be adjusted using air pressure. In this study, as shown in Figure 6b, an air pressure of 6 bar was used at a distance of approximately 1 m in the vertical direction to the LiDAR window to simulate the impact of an oncoming insect while driving.

### 2.3. Target LiDAR and Area Calculation S/W

#### 2.3.1. Target LiDAR Sample

**Figure 7:**
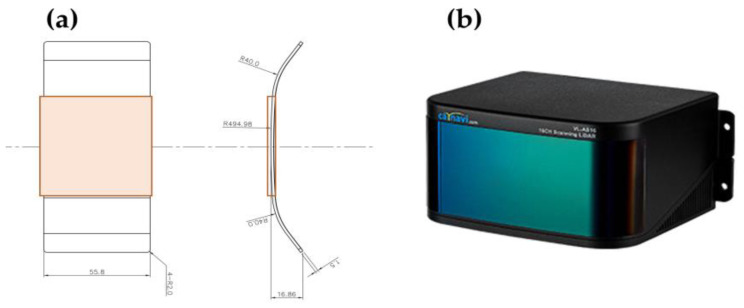
(**a**) Window sample cover dimension; (**b**) target LiDAR (VL-R16).

The selected target sample for this study, CARNAVICOM’s (13-25, Songdogwahak-ro 16beon-gil, Yeonsu-gu, Incheon, Republic of Korea) VL-R16 LiDAR window (Figure 7), includes a hard-coated window made of polycarbonate (PC) material and easily provides the blockage area unit spray amount and cleaning area.

#### 2.3.2. Image Analyzer

**Figure 8:**
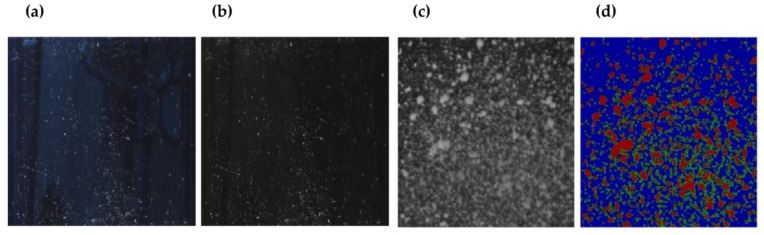
(**a**) LiDAR steady state: blue value = 53–58, (**b**) state of blue value = 0, (**c**) excluding reflected light, (**d**) RGB conversion.

As shown in Figure 8a,b, blockage tests were performed on an actual LiDAR—generating large variations in reception strength depending on the location of the blockage—the operating method of the LiDAR, and the location of the transmitter/receiver. As we focus on the blockage effect, a LiDAR sample cover was used for repeated investigations with identical conditions. In addition, the cleaning rate was determined considering the LiDAR sample cover’s area within the orange box, where the surface is free of any curves and is completely flat, as shown in Figure 7a. The concentration or area of the blockage covering the LiDAR cannot be manually analyzed because of its irregularity. Thus, automatic analysis software is essential. The cleaning surface area was calculated using Python as follows. An external light source was used to accentuate the contaminants on the LiDAR cover to capture an RGB image to the clearest extent possible. To reduce the effect of exposure to strong blue light on the clean cover, the blue value was subtracted from the RGB formula, as shown in Figure 8a,b. Although the blue value is included in the affected area, it is to a negligible degree, enabling the affected and clean areas to be distinguished. To quantify the leftover affected area, hue saturation lightness was used in place of RGB where L is the only unit used as the lightness value, as shown in Figure 8c. To identify the cleaning area on the actual LiDAR cover, various concentrations of blockage were sprayed onto the surface. Moreover, the signal strength was measured based on the LiDAR point cloud. Different blockage degrees were assigned to normal, thin, and thick blockages. As shown in Figure 8d, by comparing LiDAR point cloud values and images with blockage sprayed, we found values for the boundaries of the three areas.

### 2.4. Viscometer

**Figure 9:**
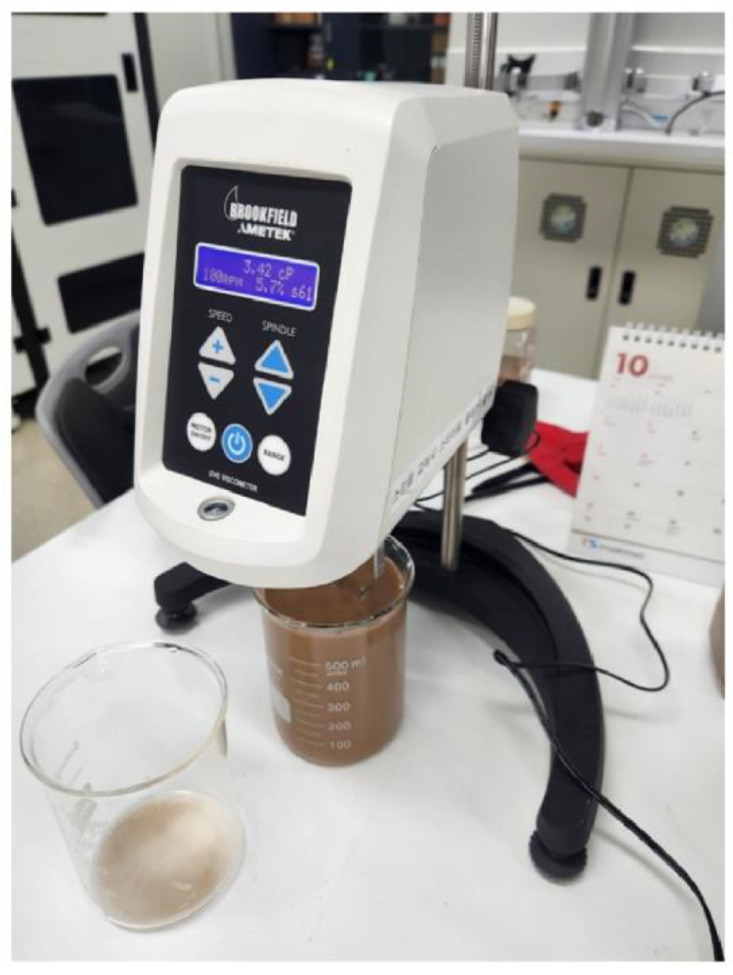
DVE viscometer.

The viscosity of blockage is significantly affected by the blockage type and concentration [38]. The viscosity of the main samples (ARI, JIS, KTD, KL, PT, FP) was measured to confirm the correlation between the blockage and the adhesion to the LiDAR window surface and the cleaning rate [38,39]. This was achieved by utilizing Brookfield’s DVE viscometer in Figure 9, which is a rotational measurement type.

## 3. Experimental Conditions and Methods

### 3.1. Characteristics of Major Factors

#### 3.1.1. Definition of Variables

For the performance comparison according to the blockage type and recipe, the manipulated variables consist of blockage type, water concentration, and dryness condition, as listed in Table 2. After spraying blockage, drying was performed for 5 min on all samples using a heating device (manufacturer: BOKUK, model name: BKH-1581PF, power: 1500 W). Because the spraying devices from different manufacturers use different washer pressure, air pressure, and duration, this study focused on blockage performance rather than setting specific pressure or duration values. To compare with cases where the blockage effect was lowered because the concentration was low, the washer pressure was 0.5 bar for 1 s of exposure and the air pressure was 2.0 bar for 1 s of exposure. The cleaning rate, which measures the blockage area remaining after washing, was selected as a dependent variable.

#### 3.1.2. Design of Experiment

The variables mentioned in Section 3.1.1 and the entire experimental progress checklist are shown in Table 3. 

### 3.2. Viscosity Analysis

**Figure 10:**
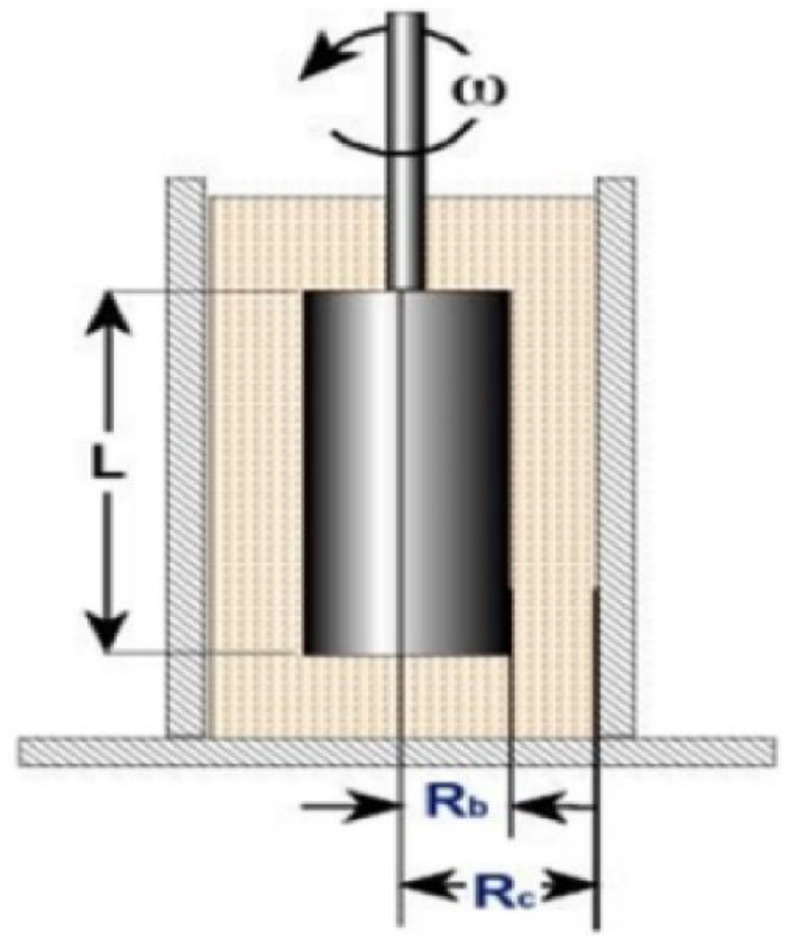
Viscometer dimension.

The main equations required for calculating viscosity through the viscometer are as follows [40]: (The configuration of the viscometer is shown in Figure 10).

(1) $$\;\mathrm{Viscosity}=\frac{Shear\;stress}{Shear\;rate},$$

(2) Shear stressdynescm2=M2πRLb2,

(3) $$Shear\;rate(s^{-1})=\frac{2\omega R_{c}{}^{2}R_{b}{}^{2}}{R_{b}{}^{2}\left(R_{c}{}^{2}-R_{b}{}^{2}\right)}\;$$

As shown in Table 4, at low viscosity a 61 spindle was used, and 62 and 64 spindles were used at high viscosity. The viscosity was measured by applying the same spindle rotation speed of 60 rpm for all concentrations. The specifications of each spindle are shown in Table 4. Viscosity values were derived using the principle of viscometer operation.

### 3.3. Methods of Experiment 

#### 3.3.1. Experimental Data Collection and Analysis Procedure 

**Figure 11:**
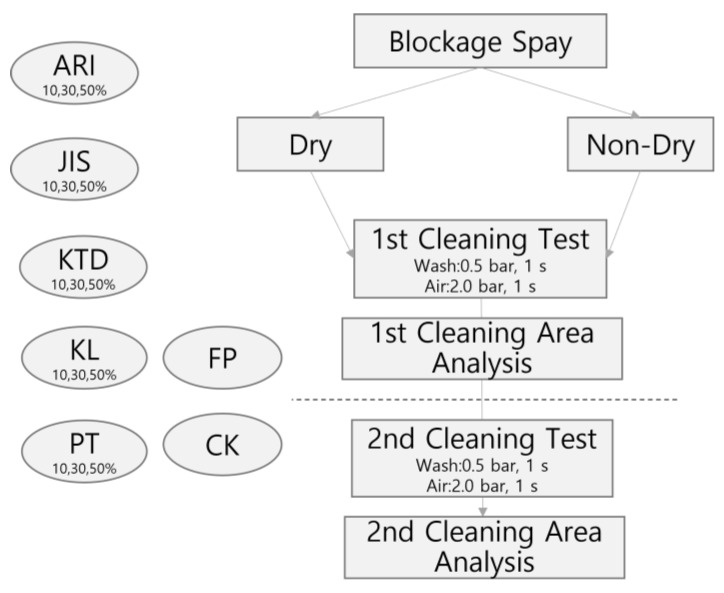
Blockage test flow chart.

**Figure 12:**
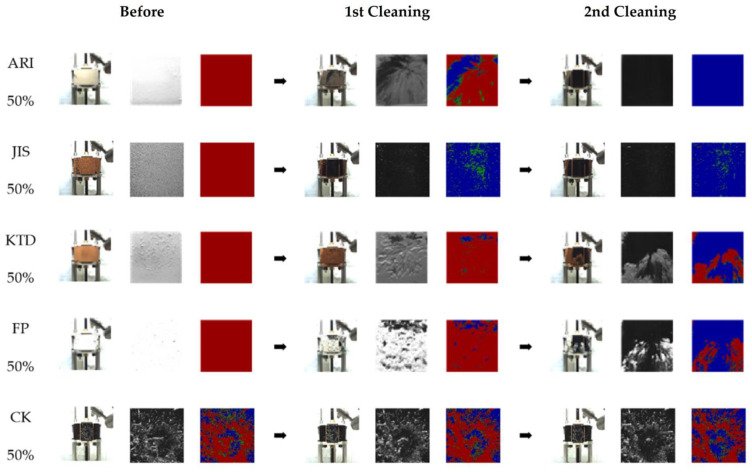
Images of the experimental process according to the type of dust (ARI, JIS, KTD, FP, CK). Items are arranged in order according to the stages of before washing, first washing, and second washing. The image of each item is expressed using a real photo, black-and-white photo, and photo of the washing area expressed in RGB.

As seen in Figure 11, the blockage sample was applied to the LiDAR window. After acquiring a photograph before washing, the first and second washings were sequentially performed, and all data were saved as photographs. The cleaning rate was calculated by checking the remaining blockage area using an image analyzer. Figure 12 shows the process of using various types of blockage for the experiment.

#### 3.3.2. Repeat Test

In this study, 3.5 g of blockage was equally applied for each item and measurements were repeated three times. A similar trend was confirmed for the same samples. Furthermore, as a result of calculating the standard deviation and standard error to confirm the similarity of the three repeated tests, the average standard deviation of the repeated tests in all items was 2.03%, and the standard error was 1.43%, which mostly fell within the confidence range.

#### 3.3.3. Data Validation Method

**Figure 13:**
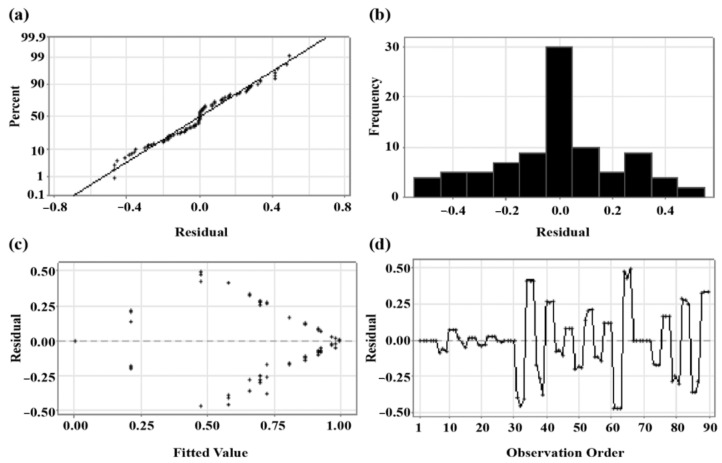
(**a**) Normal probability plot, (**b**) histogram, (**c**) versus fits, and (**d**) versus order.

Figure 13 shows ANOVA, a method to verify the mean difference between groups. The assumptions of a single ANOVA include normality, equal variance, and independence. To compare the combined means of each level, checking whether the assumptions of the ANOVA are satisfied is necessary. Residual is the value subtracted from the appropriate fitted value in the model of the observed value that was actually tested. The residual plot is a graph that checks whether these assumptions are satisfied. This is a suitable model as there is no singularity in the data distribution, and normality assumption is met.

#### 3.3.4. Random Forest 

To demonstrate the importance of the factors used in the study on the cleaning rate, the random forest algorithm was applied to assign numerical values of statistical importance to each factor.

The random forest algorithm performance is mathematically represented by the IncNodePurity value that is calculated through a series of formulas. First, the Gini importance of each node of a single decision tree within the random forest is calculated:

(4) $$G=n_{x}I_{x}-n_{l}I_{l}-n_{r}I_{r},$$

where x represents the parent node, l represents the left child node, and r represents the right child node. In addition, n represents the number of samples, and I represents the Gini impurity.

To calculate the Gini impurity, the following formula is used:

(5) $$I=1-(p_{l}^{2}+p_{r}^{2}),$$

where $p_{l}$ represents the probability of data within the parent node classifying into the left child node, whereas $p_{r}$ represents the probability of data within the parent node classifying into the right child node.

Once all nodes are assigned their respective Gini importance values, the importance of each feature on a single decision tree can be calculated by

(6) $$F=\frac{\sum_{i}G_{i}}{\sum_{j}G_{j}},$$

where $G_{i}$ represents the Gini importance of a specific feature on a singular node, whereas $G_{j}$ represents the Gini importance of all nodes.

Finally, as there are multiple decision trees associated with the random forest algorithm, the final feature importance, IncNodePurity, can be calculated as follows:

(7) $$R=\frac{\sum_{j}F_{j}}{T},$$

where $F_{j}$ represents the sum of the feature importance for each tree, and T is the total number of trees used for the random forest algorithm.

Thus, the features with the highest R value, or similarly the IncNodePurity, are classified so as to have higher overall importance as a feature in the random forest algorithm.

#### 3.3.5. Two-Way ANOVA 

The assumptions for two-way ANOVA are as follows:

- Normally distributed dependent values
- Independent samples
- Homogeneity of variances

The normality assumption can be tested by evaluating the QQ plot and conducting the Shapiro test, where a *p*-value > 0.05 confirms normality. Another important assumption that can overrule the normality assumption is the homogeneity of variances assumption, which can be made with Levene’s test. Obtaining a *p*-value greater than 0.05 confirms that the data exhibits equal variances. Though certain blockages are not normally distributed, all have equal variances; therefore, the Two-way ANOVA test can be conducted [41].

## 4. Result and Discussion

### 4.1. Importance Analysis of Major Factors Using Random Forest

**Figure 14:**
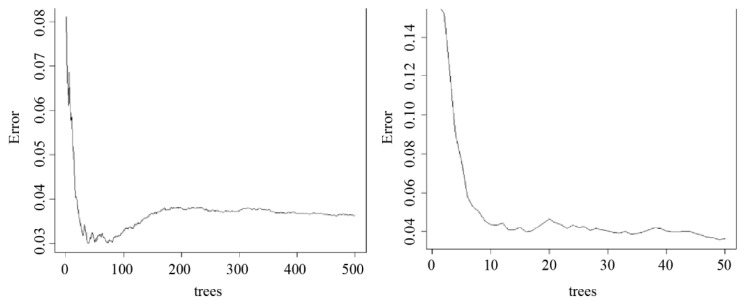
Random forest algorithm (tree case 500, 50).

Figure 14 shows that after conducting a test with 500 trees, it was determined that a random forest algorithm containing 50 trees would yield the smallest error. Thus, a random forest algorithm with 50 trees was used for the rest of the analysis discussed below.

**Figure 15:**
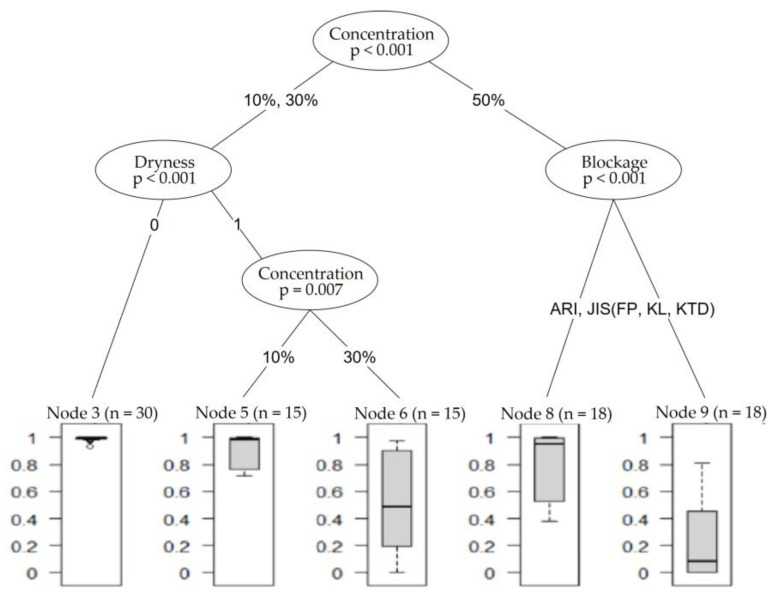
Random forest tree case.

Figure 15 shows an example of a possible singular decision tree of the 50 decision trees used to develop the random forest algorithm. 

**Figure 16:**
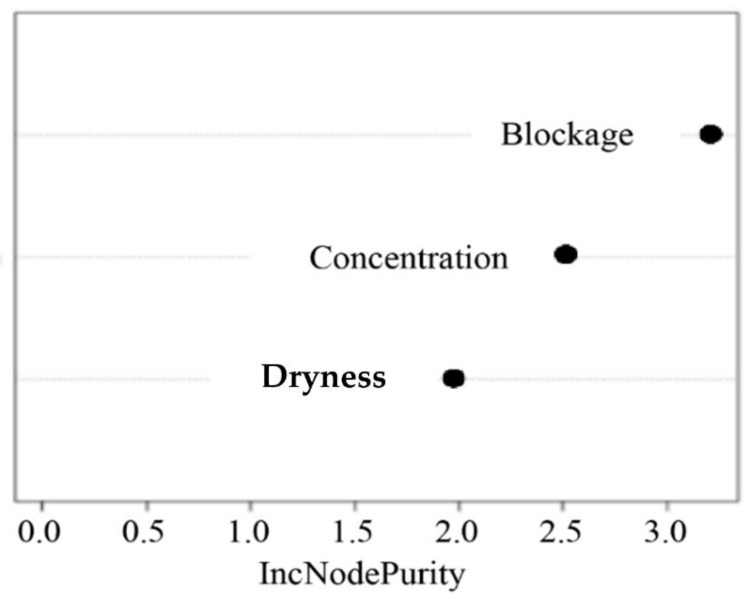
Variable importance.

From the random forest algorithm feature importance results, Table 5 shows that blockage is the most important feature with an IncNodePurity value of 3.2143. This is followed by concentration and dryness with IncNodePurity values of 2.5226 and 1.9834, respectively. The mean of squared errors is relatively low at approximately 0.0468, with a percent variance above 72%. In conclusion, Figure 16 confirms that the random forest algorithm can confidently provide the first cleaning rate using only these three variables, with blockage being the most important feature. 

### 4.2. Analysis of the Effects of Concentration and Dryness Conditions according to the Blockage Factors

Based on the results of Section 4.1, among the three factors, blockage, concentration, and dryness, the impact analysis on concentration and dryness was conducted based on the blockage factor, which is the most important factor in the cleaning rate. The two-way ANOVA technique was used to facilitate the analysis of two factors—concentration and dryness.

#### 4.2.1. ARI (Arizona Dust A2 Fine)

**Figure 17:**
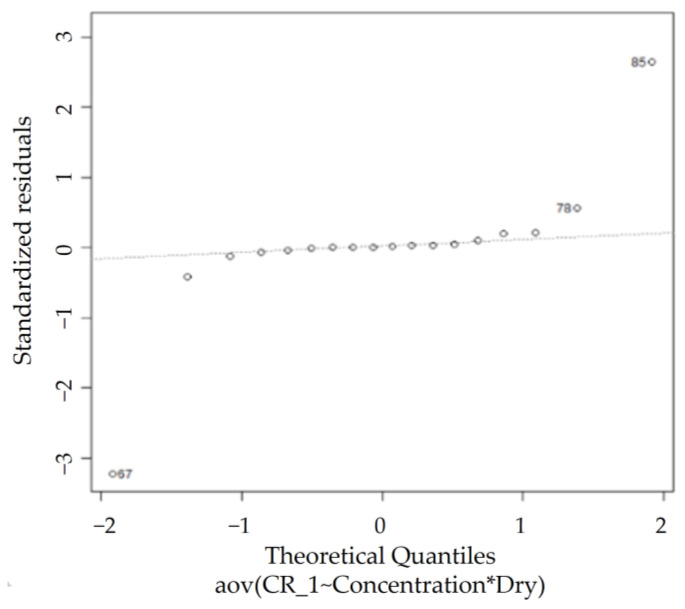
ARI normal QQ plot.

In Figure 17, according to the QQ plot, certain observations deviate significantly from the rest of the data, whereas the Shapiro–Wilk normality test rules the dataset as non-normal (*p* < 0.05), agreeing with the observations from the QQ plot. However, Table 6 reveals that the homogeneity of variance assumption is met, as Levene’s test for homogeneity of variance results show a *p*-value (0.0616) greater than 0.05. Thus, the two-way ANOVA test can be performed on this dataset.

In Table 7, from the two-way ANOVA test results for the ARI dataset, all p-values for concentration, dryness, and the interaction between concentration and dryness are below 0.05. This implies that concentration and dryness as well as their interaction term are significant factors for determining the cleaning rate. In addition, TukeyHSD results show a significant difference between all concentration groups’ differences in cleaning rate, barring the 10–30% difference (*p* = 0.1761). A significant difference in cleaning rates for ARI under non-dry conditions with 10% and 30% could not be observed.

#### 4.2.2. JIS (Class 8)

**Figure 18:**
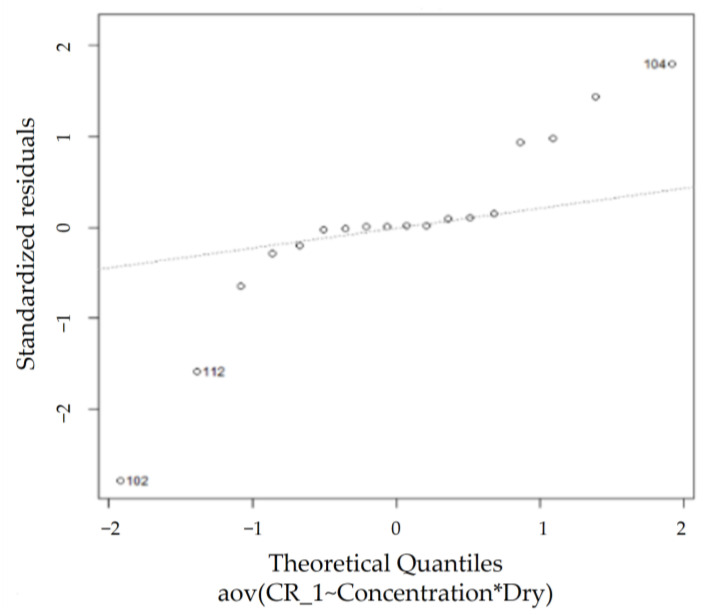
JIS normal QQ plot.

According to the QQ plot in Figure 18, certain observations deviate significantly from the rest of the data, whereas the Shapiro–Wilk normality test rules the dataset as non-normal (*p* < 0.05), agreeing with the observations from the QQ plot. However, the homogeneity of variance assumption is met as the Levene’s test for homogeneity of variance results show a *p*-value (0.3061) greater than 0.05 (shown in Table 8). Thus, the two-way ANOVA test can be performed on this dataset.

From the two-way ANOVA test results for the JIS dataset in Table 9, all *p*-values for concentration, dryness, and the interaction between concentration and dryness are below 0.05. This implies that concentration and dryness as well as their interaction term are significant factors for determining the cleaning rate. In addition, TukeyHSD results exhibit a significant difference between all concentration groups’ differences in the cleaning rate, barring a 10–30% difference (*p* = 0.2833). JIS, similar to ARI, shows an insignificant difference in the cleaning rate between non-dry conditions at 10% and 30% concentration levels.

#### 4.2.3. KTD

**Figure 19:**
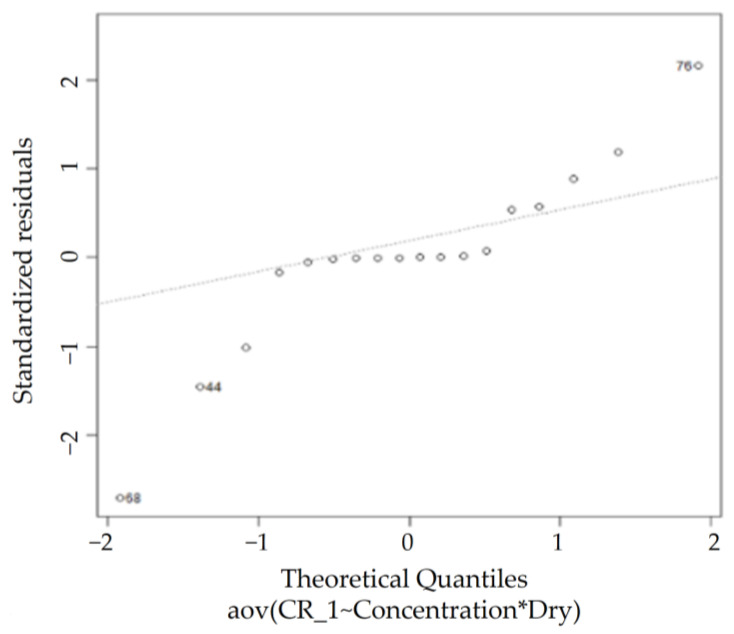
KTD normal QQ plot.

According to the QQ plot in Figure 19, certain observations that deviate significantly from the rest of the data are observed, whereas the Shapiro–Wilk normality test rules the dataset as non-normal (*p* < 0.05), agreeing with the observations from the QQ plot. However, the homogeneity of variance assumption is met, as the Levene’s test for homogeneity of variance results exhibits a *p*-value (0.1975) greater than 0.05, as shown in Table 10. Thus, conducting the two-way ANOVA test on this dataset is feasible.

From the two-way ANOVA test results for the KTD dataset in Table 11, all *p*-values for concentration, dryness, and the interaction between concentration and dryness are below 0.05. This implies that concentration and dryness as well as their interaction terms are significant factors for determining the cleaning rate. In addition, TukeyHSD results show a significant difference between all concentration groups’ differences (*p* < 0.05) in the cleaning rate.

#### 4.2.4. KL (Kaolin)

**Figure 20:**
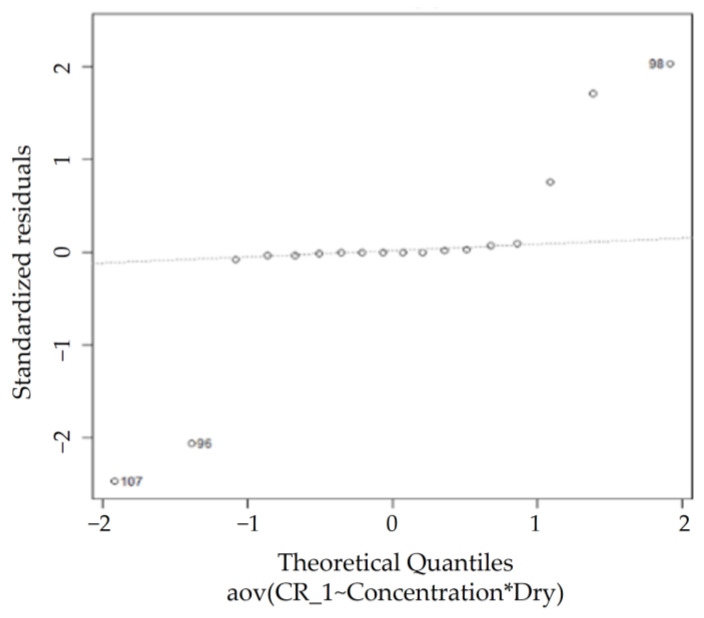
KL normal QQ plot.

According to the QQ plot in Figure 20, certain observations that deviate significantly from the rest of the data are observed, whereas the Shapiro–Wilk normality test rules the dataset as non-normal (*p* < 0.05), agreeing with the observations from the QQ plot. However, the homogeneity of variance assumption is met, as the Levene’s test for homogeneity of variance results show a *p*-value (0.1324) greater than 0.05, as shown in Table 12. Thus, performing a two-way ANOVA test on this dataset is feasible.

From the two-way ANOVA test results for the KL dataset in Table 13, all *p*-values for concentration, dryness, and the interaction between concentration and dryness are below 0.05. This implies that concentration and dryness as well as their interaction term are significant factors for determining the cleaning rate. In addition, Tukey HSD results exhibit a significant difference between all concentration groups’ differences (*p* < 0.05) in the cleaning rate.

#### 4.2.5. Portland Cement (PT)

**Figure 21:**
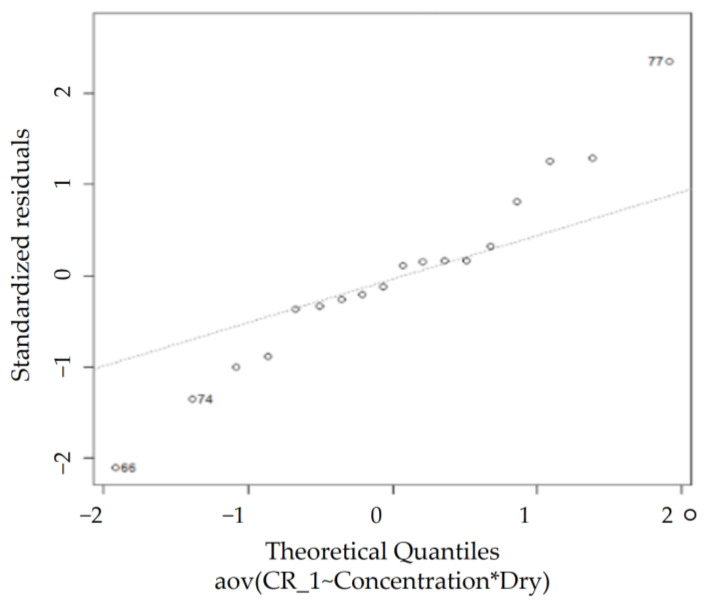
PT normal QQ plot.

According to the QQ plot and the Shapiro–Wilk normality test in Figure 21, the data are observed to be normal (*p* > 0.05). In addition, the homogeneity of variance assumption is also met, as the Levene’s test for homogeneity of variance results show a *p*-value (0.6425) greater than 0.05, as shown in Table 14. Thus, performing the two-way ANOVA test on this dataset is feasible.

From the two-way ANOVA test results for the PT dataset in Table 15, all *p*-values for concentration, dryness, and the interaction between concentration and dryness are below 0.05. This implies that concentration and dryness as well as their interaction term are significant factors for determining the cleaning rate. In addition, Tukey HSD results show a significant difference between all concentration groups’ differences (*p* < 0.05) in the cleaning rate.

### 4.3. Comparison of Cleaning Rates according to Blockage, Concentration, and Dryness Conditions

**Figure 22:**
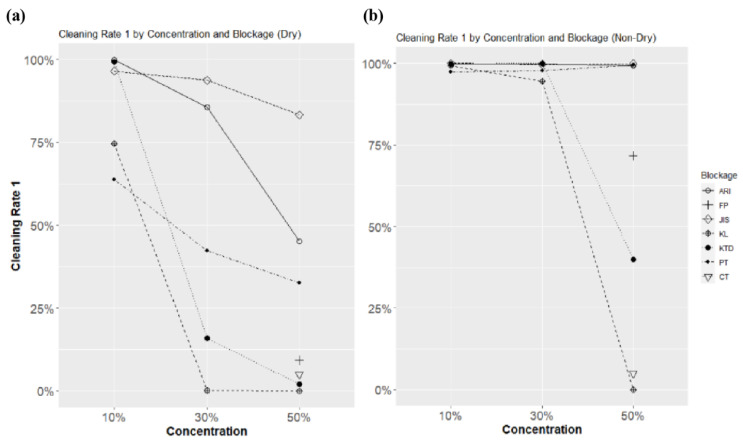
(**a**) Blockage and concentration cleaning rate result graph (dry), (**b**) blockage and concentration cleaning rate result graph (non-dry).

Figure 22a shows the cleaning rate with respect to concentration for all blockage factors under dry conditions. The characteristics of each blockage were apparent, and various trends in the cleaning rate change according to the concentration of each blockage factor were revealed. JIS and PT showed negligible changes in the cleaning rate by concentration, and ARI showed an apparent change in the cleaning rate in all concentration ranges. Particularly for KTD and KL, the change in concentration was considerably large, from 10% to 30%, and the change in the cleaning rate from 30% to 50% concentration was relatively small at 13.89% for KTD and 0.14% for KL. This is because the cleaning condition, which is an independent variable of this experimental condition, is insufficient. The FT and CK values, which are comparative indicators, fall within the 30% and 10% ranges of KTD, respectively. In the future, when considering the influence of bird droppings or insects, equivalent performance is expected within the 30–50% concentration range of KTD. Finally, the cleaning rate of KL was exceedingly low at concentrations above 30%; thus, comparison with other factors is irrelevant.

Figure 22b shows the cleaning rate by concentration for all blockage factors under non-drying conditions. All cleaning rates were high compared with dry conditions. In the range of 10–30%, all blockage cleaning rates showed high values of 94.53% or more. However, ARI, JIS, and PT did not affect the concentration change, and only KTD and KL showed a sharp decrease in the cleaning rate in the 50% range. KL did not exhibit clear characteristics under the cleaning conditions used in this study at 50% concentration, even under non-dry conditions. Thus, KL exhibited a higher performance as a blockage than other factors, and accurate distinction is difficult when employing the fixed washing performance in this study.

The overall performance was verified based on the above results. Compared with the performances of FP and CK, which are comparative indicators, blockages with appropriate performance can be created by utilizing the cleaning rate result according to the concentrations of KTD and PT depending on dry or non-dry conditions.

**Figure 23:**
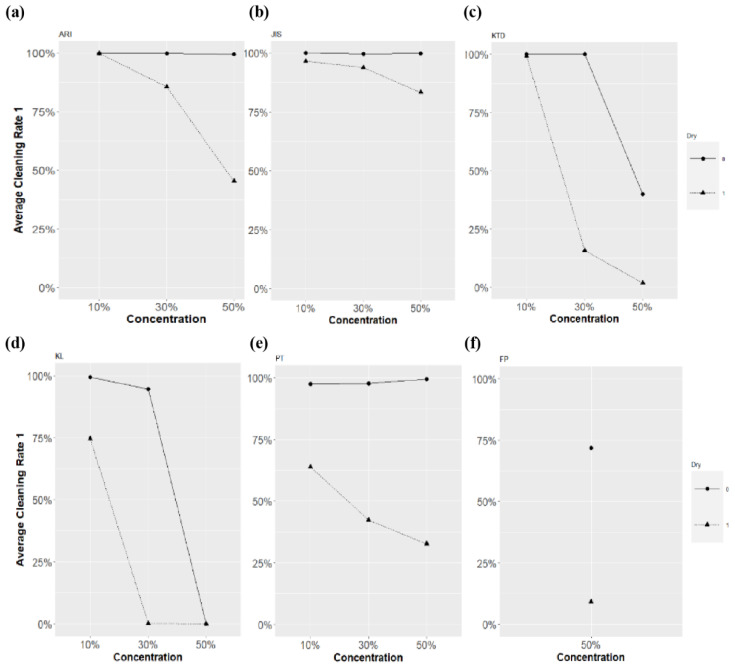
Comparison of dry and non-dry characteristics of blockages (**a**) ARI, (**b**) JIS, (**c**) KTD, (**d**) KL, (**e**) PT, (**f**) FP.

Figure 23 compares the dry and non-dry characteristics of each blockage factor. Regardless of whether the environment was dry, ARI, JIS, and KTD demonstrated a cleaning rate >96.52% at 10% concentration. However, most of the dry and non-dry cleaning rates clearly differed at 30% concentration. In the cases of KTD, KL, and PT, a noticeable variation in the cleaning rate was observed. Furthermore, concentration and drying circumstances impact these results because the cleaning rate, which ranges from 10% to 30% under comparatively non-dry conditions, is marginally changed. Because the cleaning rate immediately dropped to almost zero in a non-dry state at a concentration of 50%, KL could not validate the drying characteristics. According to FP, a significant difference of 62.47% in the cleaning rate between dry and non-dry conditions was noted.

### 4.4. Comparison of Characteristics according to Cleaning Cycles 

This study compared the performance according to the blockage type, concentration, and dryness condition based on the constant cleaning performance. However, the following graph compares the cleaning rate results under two repetitions of the same cleaning performance, as discussed below.

**Figure 24:**
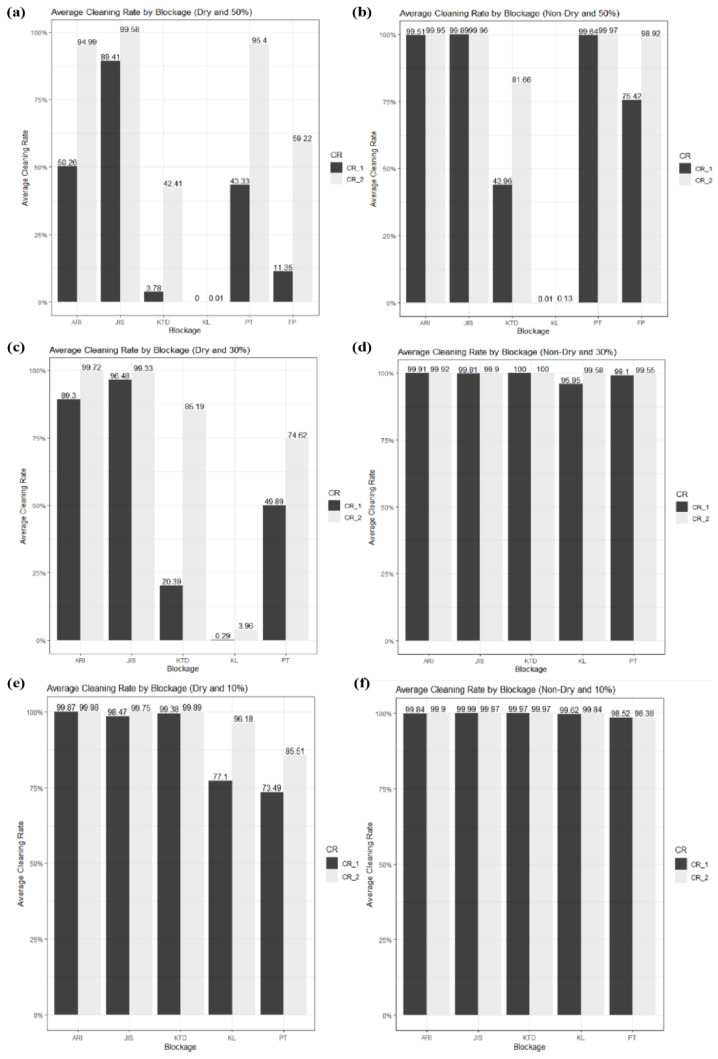
Difference between first and second cleaning rates. (**a**) Concentration 50%, first cleaning rate; (**b**) 50%, second cleaning rate; (**c**) 30%, first cleaning rate; (**d**) 30%, second cleaning rate; (**e**) 10%, first cleaning rate; and (**f**) 10%, second cleaning rate.

In the comparison of the difference between the first and second cleaning rates in Figure 24, most factors with a large difference arose from a concentration of 30% or more. Only KL and PT were affected at 10% concentration. Various results can be obtained from the graph, depending on the washing performance (e.g., amount of water, spray pressure, time). In particular, the cleaning rate increased rapidly when KTD, PT, and FP at 50% concentration and KTD, KL, and PT at 30% concentration were washed twice. 

### 4.5. Correlation between Viscosity and Cleaning Rate

To identify the correlation between viscosity and cleaning rate and to compare the viscosity and cleaning rate values, the viscosity values were measured using a viscometer for each blockage and concentration, as shown in Table 16.

The correlation between the viscosity and cleaning rate was analyzed. The closer the correlation is to zero, the weaker the connection between the viscosity and cleaning rate. A negative value was obtained when the viscosity and cleaning rate were high and low, respectively. Conversely, a positive value was obtained when both the cleaning rate and viscosity were high.

**Figure 25:**
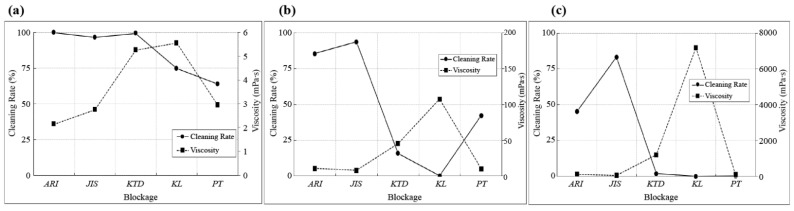
Viscosity versus cleaning rate comparison graph. (**a**) Concentration 10%; (**b**) concentration 30%; and (**c**) concentration 50%.

The negative correlation coefficient in the dry state indicates that the viscosity and cleaning rate are inversely correlated, as shown in Table 17. In particular, the correlation tendency was clearly confirmed at 30 and 50% concentrations under dry conditions. However, under the dry condition of 10% and the non-dry condition of 10 and 30%, the cleaning rate by blockage factor is mostly high at 90% or more; thus, the correlation tendency is unclear. Therefore, the correlation with viscosity is not large because the correlation coefficient with viscosity is close to 0. Although the results were not satisfactory under all conditions, Figure 25 shows that the correlation between viscosity and cleaning rate was high under dry conditions, and a large correlation was observed only at 50% concentration under non-dry conditions. If the viscosity is high, cleaning is difficult because the blockage strongly adheres to the LiDAR window sample. Moreover, this adhesion can be effective in selecting suitable parameters for spray testing. KTD and KL are efficient for cleaning tests because a large amount of dust can adhere to a small amount of the target sample.

## 5. Conclusions

Based on the cleaning rate, this study examined how various factors, such as the blockage type, concentration, additive, and dryness, which are the main factors in the evaluation of the cleaning performance of autonomous driving sensors, affect blockage performance. The findings of this study can be applied to various blockages, and blockages with the desired performance, such as bird droppings and insects, can be implemented by adjusting the dryness and concentration. 

KTD dust, representing the Korean environment, was presented, and the cleaning rate was compared in addition to ARI and JIS. At a concentration of 30% or higher, KTD exhibited a considerably lower cleaning rate than ARI and JIS. The effect of KTD as a substitute dust was similar to that of bird droppings as the cleaning rate for KTD was within the range similar to that of the cleaning rate for bird droppings. Experimental tools and techniques for performance testing were proposed in this study to compare the performance of dust in a particular region in the future. Therefore, it will be possible to increase the variety of dust options, lower the price of dust purchases, and represent the actual environment as closely as possible.

To evaluate the sensor cleaning performance for the safe autonomous driving of SAE level four or higher, experiments can be conducted under various conditions by using various recipes (e.g., type, concentration, drying) suggested in this study. Furthermore, they can also be used for various sensor cleaning evaluations by utilizing methods such as cross-mixing and the five blockages covered in this study, even in severe cases, such as bird droppings and insects, which are frequently encountered during actual road driving.

Finally, the number of washings had a substantial effect on the cleaning rate. Further research is required to optimize the cleaning technology through a comparative test of the cleaning rate for blockages, such as KTD, KL, FP, and CK, under severe conditions with the cleaning conditions, such as water and air injection pressure and time, fixed to a low specification as in this study and used as independent variables. Furthermore, the blockage effect on camera and radar sensors and LiDAR sensors and the optimal cleaning conditions for each sensor need to be researched. This method can be used for LiDAR, which is the target sensor of this study, and also cameras, radars, and ultrasonic sensors. It can be widely used in various tests involving devices such as automobile windshields, mirrors, and license plates.

## Figures and Tables

**Table 1:** Design specification.

Dust	Particle Size	Ingredients	Nation
ARI A2Fine	Under 88 μm	SiO_2_, Al_2_O_3_, Fe_2_O_3_, Na_2_O	USA
JIS Class 8	Under 75 μm	SiO_2_, Al_2_O_3_, Fe_2_O_3_, Na_2_O, MgO, CaO	Japan
KTD	Under 85 μm	SiO_2_, Al_2_O_3_, Fe_2_O_3_, Na_2_O, CaO, MgO, TiO_2_, K_2_O	Republic of Korea

**Table 2:** Main factors of the experiment.

Type	Variable
Manipulated variable	Blockage (ARI, JIS, KTD, kaolin, Portland cement, fake poop, insect), concentration (10%, 30%, and 50%), dryness condition
Control variable	Window, washer: 0.5 bar for 1 s, air: 2.0 bar for 1 s
Dependent variable	Cleaning rate (%)

**Table 3:** Experimental progress checklist.

Blockage	Concentration			Dry	Non Dry	Note
	10%	30%	50%			
ARI	√	√	√	√	√	
JIS	√	√	√	√	√	
KTD	√	√	√	√	√	
KL	√	√	√	√	√	
PT	√	√	√	√	√	
FP	-	-	√	√	√	Concentration of 50% only
CK	-	-	-	-	√	Rate is not distinguishable. Immediately performed without drying

Tests were performed under dry and non-dry conditions at concentrations of 10%, 30%, and 50% for ARI, JIS, KTD, kaolin (KL), and Portland cement (PT). In the case of fake poop (FP), a single concentration of 50% was selected, which exhibits the most similar concentration to that of bird poop in the real environment. In the field of insects, a cricket (CK) was used and the cleaning test was conducted immediately after firing on the sensor surface.

**Table 4:** Viscosity value measurement variable, spindle specification.

Variable	Spindle		
M = Torque acting on surface of spindle	<61 spindle>	<62 spindle>	<64 spindle>
ω = Angular velocity of spindle (radians/s) = 2π/60 × N where N = spindle RPM	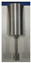	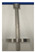	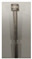
Rc = Radius of container Rb = Radius of spindle L = Effective spindle side length	Rb = 10mm,	Rb = 10 mm,	Rb = 2 mm,
	Rc = 42.5 mm	Rc = 42.5 mm,	Rc = 42.5 mm,
	L = 65 mm	L = 8 mm,	L = 33 mm,
	N = 60 rpm	N = 60 rpm	N = 60 rpm

**Table 5:** Evaluation of the factor importance of the random forest model.

Factor	Importance	*p*
Blockage	3.2143	0.000
Concentration	2.5226	0.000
Dry	1.9834	0.000
Mean of squared residuals	03624	
% Var explained	72.69	

**Table 6:** Shapiro and Levene’s test result (ARI).

Shapiro–Wilk Normality Test	
w	0.6274
*p*	0.0000
Levene’s Test for Homogeneity of Variance	
diff	5
F-value	2.8854
*p*	0.0616

**Table 7:** Summary of factor importance and Tukey HSD results (ARI).

Summary	Df	Sum sq	Mean sq	F Value	*p*
Concentration	2	0.2079	0.1040	46.21	0.000
Dryness	1	0.1790	0.1794	79.58	0.000
Concentration * Dryness	2	0.2018	0.1010	44.84	0.000
Residuals	12	0.0270	0.0023	-	-
TukeyHSD	Tukey multiple comparisons of means 95% family-wise confidence level				
	diff	lwr	upr	*p*	
30−10%	−0.0525	−0.1256	0.0205	0.1761	
50−10%	−0.2497	−0.3227	−0.1766	0.0000	
50−30%	−0.1972	−0.2702	−0.1241	0.0000	

**Table 8:** Shapiro and Levene’s test result (JIS).

Shapiro–Wilk Normality Test	
w	0.8748
*p*	0.0213
Levene’s Test for Homogeneity of Variance	
diff	5
F-value	1.3584
*p*	0.3061

**Table 9:** Summary of factor importance and Tukey HSD results (JIS).

Summary	Df	Sum sq	Mean sq	F Value	*p*
Concentration	2	0.0069	0.0034	25.17	0.000
Dryness	1	0.0117	0.0117	86.13	0.000
Concentration * Dryness	2	0.0067	0.0034	24.67	0.000
Residuals	12	0.0016	0.0001	-	-
Tukey HSD	Tukey multiple comparisons of means 95% family-wise confidence level				
	diff	lwr	upr	*p*	
30−10%	−0.0108	−0.0288	0.0072	0.2833	
50−10%	−0.0458	−0.0637	−0.0278	0.0000	
50−30%	−0.0350	−0.0530	−0.0170	0.0006	

**Table 10:** Shapiro and Levene’s test result (KTD).

Shapiro–Wilk Normality Test	
w	0.8813
*p*	0.0274
Levene’s Test for Homogeneity of Variance	
diff	5
F-value	1.7520
*p*	0.1975

**Table 11:** Summary of factor importance and Tukey HSD results (KTD).

Summary	Df	Sum sq	Mean sq	F value	*p*
Concentration	2	1.7249	0.8625	975.4	0.000
Dryness	1	0.7246	0.7246	819.5	0.000
Concentration * Dryness	2	0.4684	0.2342	264.9	0.000
Residuals	12	0.0106	0.0009	-	-
Tukey HSD	Tukey multiple comparisons of means 95% family-wise confidence level				
	diff	lwr	upr	*p*	
30−10%	−0.3948	−0.4406	−0.3490	0.0000	
50−10%	−0.7581	−0.8039	−0.7122	0.0000	
50−30%	−0.3632	−0.4090	−0.3174	0.0006	

**Table 12:** Shapiro and Levene’s test result (KL).

Shapiro–Wilk Normality Test	
w	0.7566
*p*	0.0004
Levene’s Test for Homogeneity of Variance	
diff	5
F-value	2.123
*p*	0.1324

**Table 13:** Summary of factor importance and Tukey HSD results (KL).

Summary	Df	Sum sq	Mean sq	F Value	*p*
Concentration	2	2.3482	1.1741	5975	0.000
Dryness	1	0.6984	0.6984	3555	0.000
Concentration * Dryness	2	0.7504	0.3752	1910	0.000
Residuals	12	0.0024	0.0002	-	-
Tukey HSD	Tukey multiple comparisons of means 95% family-wise confidence level				
	diff	lwr	upr	*p*	
30−10%	−0.4024	−0.4240	−0.3808	0.0000	
50−10%	−0.8836	−0.9051	−0.8620	0.0000	
50−30%	−0.4812	−0.5028	−0.4596	0.0006	

**Table 14:** Shapiro and Levene’s test result (PT).

Shapiro–Wilk Normality Test	
w	0.9698
*p*	0.7941
Levene’s Test for Homogeneity of Variance	
diff	5
F-value	0.6871
*p*	0.6425

**Table 15:** Summary of factor importance and Tukey HSD results (PT).

Summary	Df	Sum sq	Mean sq	F Value	*p*
Concentration	2	0.0705	0.0352	102.5	0.000
Dryness	1	0.8523	0.8523	2480.1	0.000
Concentration* Dryness	2	0.0807	0.0404	117.5	0.000
Residuals	12	0.0041	0.0003	-	-
Tukey HSD	Tukey multiple comparisons of means 95% family-wise confidence level				
	diff	lwr	upr	*p*	
30−10%	−0.1151	−0.1436	−0.0865	0.0000	
50−10%	−0.1452	−0.1737	−0.1166	0.0000	
50−30%	−0.0587	−0.0587	−0.0016	0.0386	

**Table 16:** Viscosity result for each blockage (Unit: mPa·s).

Blockage	Blockage Concentration			Blockage	Blockage Concentration		
	10%	30%	50%		10%	30%	50%
ARI	2.15	10.55	118.25	KL	5.55	107.50	7185
JIS	2.75	7.95	57.25	PT	2.95	10.15	95
KTD	5.25	45.45	1205	FP	-	-	160

**Table 17:** Correlation between cleaning rate and viscosity.

Concentration (ARI, JIS, KTD, KL, PT)	Dry	Non-Dry
10%	−0.149	0.232
30%	−0.822	0.289
50%	−0.629	−0.904

## Data Availability

Not applicable.
